# Axonal Localization of Ca^2+^-Dependent Activator Protein for Secretion 2 Is Critical for Subcellular Locality of Brain-Derived Neurotrophic Factor and Neurotrophin-3 Release Affecting Proper Development of Postnatal Mouse Cerebellum

**DOI:** 10.1371/journal.pone.0099524

**Published:** 2014-06-12

**Authors:** Tetsushi Sadakata, Wataru Kakegawa, Yo Shinoda, Mayu Hosono, Ritsuko Katoh-Semba, Yukiko Sekine, Yumi Sato, Chihiro Saruta, Yasuki Ishizaki, Michisuke Yuzaki, Masami Kojima, Teiichi Furuichi

**Affiliations:** 1 Advanced Scientific Research Leaders Development Unit, Gunma University, Maebashi, Gunma, Japan; 2 JST/CREST, Kawaguchi, Saitama, Japan; 3 Department of Physiology, School of Medicine, Keio University, Tokyo, Japan; 4 Department of Applied Biological Science, Tokyo University of Science, Noda, Chiba, Japan; 5 Department of Molecular and Cellular Neurobiology, Gunma University Graduate School of Medicine, Maebashi, Gunma, Japan; 6 Biointerface Research group, Health Research Institute, National Institute of Advanced Industrial Science and Technology (AIST), Ikeda, Osaka, Japan; Institut National de la Santé et de la Recherche Médicale (INSERM U901), France

## Abstract

Ca^2+^-dependent activator protein for secretion 2 (CAPS2) is a protein that is essential for enhanced release of brain-derived neurotrophic factor (BDNF) and neurotrophin-3 (NT-3) from cerebellar granule cells. We previously identified dex3, a rare alternative splice variant of CAPS2, which is overrepresented in patients with autism and is missing an exon 3 critical for axonal localization. We recently reported that a mouse model CAPS2^Δex3/Δex3^ expressing dex3 showed autistic-like behavioral phenotypes including impaired social interaction and cognition and increased anxiety in an unfamiliar environment. Here, we verified impairment in axonal, but not somato-dendritic, localization of dex3 protein in cerebellar granule cells and demonstrated cellular and physiological phenotypes in postnatal cerebellum of CAPS2^Δex3/Δex3^ mice. Interestingly, both BDNF and NT-3 were markedly reduced in axons of cerebellar granule cells, resulting in a significant decrease in their release. As a result, dex3 mice showed developmental deficits in dendritic arborization of Purkinje cells, vermian lobulation and fissurization, and granule cell precursor proliferation. Paired-pulse facilitation at parallel fiber-Purkinje cell synapses was also impaired. Together, our results indicate that CAPS2 plays an important role in subcellular locality (axonal vs. somato-dendritic) of enhanced BDNF and NT-3 release, which is indispensable for proper development of postnatal cerebellum.

## Introduction

Ca^2+^-dependent activator protein for secretion 2 (CAPS2) is a member of the CAPS/CADPS protein family that regulates the trafficking of dense-core vesicles by binding both phosphoinositides and dense-core vesicles [1]–[5]. The human *CAPS2* gene locus (7q31.32) is located within the autism susceptibility locus 1 (AUTS1) [6] on chromosome 7q31–q33, one of several susceptibility loci for autism [7]. Mouse CAPS2 protein is associated with secretory vesicles containing brain-derived neurotrophic factor (BDNF) and is involved in promoting the activity-dependent release of BDNF [8], [9]. BDNF plays a key role in many aspects of brain development and function, including the formation of synapses and circuits [10], [11]. It has been shown that the decreased level of BDNF expression in methyl CpG-binding protein 2 (*Mecp2*)-mutant mice, a model of Rett syndrome [12], affects disease progression [13].

We previously showed that the expression of an exon 3-skipped (or -spliced out) form of CAPS2 (designated CAPS2-dex3) [14], which is now known as a rare alternative splicing variant [15], [16], is increased in a subgroup of patients with autism and is not properly transported into axons [14]. Moreover, we demonstrated that neurons with increased expression of dex3 fail to properly coordinate local BDNF release from axons [14], [15]. In addition, the association of CAPS2 with autism has been suggested not only by the presence of copy number variations in the CAPS2 gene in autistic patients [17]–[19], but also by decreased transcription of CAPS2 in the brains of people with autism [20]. Recently, it was also reported that *CAPS2* deletion and missense mutations of maternal origin contribute to onset [21].

We previously generated a mouse model CAPS2^Δex3/Δex3^ that expressed autism-associated CAPS2 isoform dex3 and revealed that dex3 mice showed the autistic-like behavioral phenotypes [22]. In this report, we analyzed the cellular and physiological phenotypes of CAPS2^Δex3/Δex3^ mice by focusing on the cerebellum and found that exon 3 skipping of CAPS2 influence subcellular locality (axon vs. somato-dendrite) of BDNF and NT-3 release, resulting in aberrant development of postnatal cerebellum.

## Materials and Methods

### Animals

CAPS2-exon3 skipped mice (deletion of exon 3, dex3) mice (CAPS2^Δex3/Δex3^) were used as described in a previous study [22]. All experimental protocols were approved by the Institutional Animal Care and Use Committee of RIKEN, Gumma University, Keio University, and Tokyo University of Science. Mice were housed on a 12:12 h light dark cycle, with the dark cycle occurring from 20:00 to 8:00.

### Antibodies

The following primary antibodies were used for immunohistochemistry: guinea pig polyclonal anti-CAPS2 (1∶4,000 dilution) [9], rabbit polyclonal anti-BDNF (1∶100 dilution) (Katoh-Semba et al., 1997), rabbit polyclonal anti-NT-3 (1∶150 dilution) [23], rabbit polyclonal anti-calbindin (1∶1,000 dilution; cat. no. AB1778, Millipore, Billerica, MA, USA), mouse monoclonal anti-tau (1∶300 dilution; cat. no. 610672; BD Biosciences, San Jose, CA), and anti-phosphorylated Trk (Tyr490) (1∶10 dilution; cat. no. 9141; Cell Signaling Technology, Beverly, MA).

### Immunohistochemistry

C57BL/6J male mice were used after being sacrificed by anesthesia with diethyl ether. Mice were transcardially perfused, initially with PBS and then with 4% PFA in PBS. Tissues were dissected out, postfixed in 4% PFA at 4°C for 5 h, and cryoprotected by immersion in 15% sucrose in PBS overnight at 4°C. After embedding in Tissue-Tek OCT compound (Sakura Finetechnical, Tokyo, Japan), tissues were frozen in dry ice powder, and sectioned at a thickness of 15 µm using a cryostat (CM1850; Leica Microsystems, Frankfurt, Germany) at −18°C. Sections were air-dried for 1 h, and rinsed three times in PBS. After blocking with 5% donkey normal serum (Vector, Burlingame, CA, USA) in PBS, sections were reacted with specific primary antibodies at 4°C overnight, rinsed in PBS, reacted with the appropriate secondary antibody at room temperature for 1 h, and again rinsed in PBS. Immunoreacted sections were mounted with Vectorshield (Vector) mounting medium, and observed using a microscope (BX51, Olympus, Tokyo, Japan) equipped with a CCD camera (VB-7000, Keyence, Osaka, Japan). Digital images were processed using Adobe Photoshop 6.0 software.

### Cerebellar primary cultures

Cerebellar primary cultures were prepared as described in a previous study [24] with minor modification. In brief, after rapid decapitation the cerebella of P0 mice were dissected out, digested for 13 minutes at 37°C with 0.1% Trypsin (Sigma-Aldrich, St. Louis, MO, USA) and 0.05% DNase I (Boehringer Mannheim, Indianapolis, IN, USA) in Ca^2+^/Mg^2+^-free Hanks' balanced salt solution [HBSS-CaMg(−); Sigma], and washed with HBSS-CaMg(−). They were then triturated by repeated passage through a 1-ml plastic micropipette tip in HBSS-CaMg(−) containing 0.05% DNase I and 12 mM MgSO_4_, and washed with a serum-free Eagle minimal essential medium-based chemical-conditioned medium supplemented with 0.25% (w/v) glucose (Nacalai Tesque, Kyoto, Japan), 10 µg/ml insulin (Sigma-Aldrich), 0.1 nM L-thyroxine (Sigma-Aldrich), 0.1 mg/ml apotransferrin (Sigma-Aldrich), 1 mg/ml bovine serum albumin (BSA; Sigma-Aldrich), 2 mM L-glutamine (Nacalai Tesque), 1 µg/ml aprotinin (Sigma-Aldrich), 30 nM sodium selenite (Merck, Darmstadt, Germany), 100 U/ml penicillin (Banyu Pharmaceutical, Tokyo, Japan), and 135 µg/ml streptomycin (Meiji Seika K.K., Tokyo, Japan). The dissociated cells were plated at 5 × 10^5^ cells per glass coverslip (12 mm in diameter; Matsunami, Tokyo, Japan), coated with poly-L-lysine (Sigma-Aldrich) and then cultured in the serum-free Eagle's minimal essential medium-based chemical-conditioned medium described above at 37 °C under a humidified 5% CO_2_ atmosphere.

### NT-3 release assay

The culture media were collected at 7 days *in vitro* (DIV). The quantity of NT-3 released into the media was measured using a highly sensitive two-site enzyme immunoassay as previously described [9], [25].

### Immunocytochemistry

Transfection was carried out using Lipofectamine 2000 Reagent (Invitrogen, Carlsbad, CA, USA). Forty-eight hours after transfection, cells were fixed with Zamboni's fixative (2% PFA in 0.1 M phosphate buffer, pH 7.4, containing 0.2% picric acid) at room temperature for 15 min. After washing three times with PBS, cells were permeabilized in PBS containing 0.02% Triton X-100 at room temperature for 5 min. After blocking with 5% normal donkey serum in PBS at room temperature for 60 min, cells were incubated with specific primary antibodies at 4°C overnight, rinsed in PBS, then incubated with Alexa-conjugated secondary antibodies (1∶1,000 dilution; Invitrogen) at room temperature for 1 h, and again rinsed in PBS. Immunoreacted cells were mounted with Vectashield (Vector) mounting medium. Images were acquired with a microscope (BX51, Olympus) equipped with a CCD camera (VB-7000, Keyence). Digital images were processed using Adobe Photoshop 6.0 software. To quantify immunoreactivity, the region of interest was analyzed using NIH ImageJ software (National Institutes of Health, Bethesda, MD).

### Electron microscopy

Under deep Nembutal anesthesia (250 mg/Kg, i.p.), P21 mice were transcardially perfused with 2 ml of saline followed by 25 ml of a mixture of 2.5% glutaraldehyde and 2% paraformaldehyde in 0.1 M PB. Brains were dissected and immersed in the same fixative at 4°C overnight. The cerebellum was cut into 300 mm transverse slices on a microslicer (VT1000S, Leica Microsystems) and post-fixed in cold 1% OsO_4_ solution for 1 h. After dehydration in a graded series of alcohol solutions, slices were embedded in epoxy resin (EPON 812, Taab Laboratories, Reading, UK). Serial ultrathin 70 nm sections were cut on an ultramicrotome (EM UC6, Leica Microsystems), mounted on 200 mesh uncoated copper grids, and metal stained with uranyl acetate/lead citrate. The grids were examined with a transmission electron microscope (1200EX, JEOL) at 80 kV.

### Electrophysiology

Parasagittal cerebellar slices (200 µm thick) were prepared from CAPS2^Δex3/Δex3^ mice and wild-type littermates (P25–35) in accordance with the institution's guidelines of Keio University, as previously reported [26]. Whole-cell voltage-clamp recordings were made from visually identified Purkinje cells (PCs) with an Axopatch 200B amplifier (Axon Instruments, Foster City, CA), and the pCLAMP system (version 9.2, Axon Instruments) was used for data acquisition and analysis. Patch pipettes were pulled from borosilicate glass capillaries to achieve a resistance of 2 to 4 MΩ when filled with a solution containing (65 mM K-gluconate, 65 mM Cs-methanesulfonate, 10 mM KCl, 1 mM MgCl_2_, 4 mM Na_2_ATP, 1 mM Na_2_GTP, 20 mM HEPES, 5 mM sucrose, and 0.4 mM EGTA (pH 7.3, 295 mOsm/kg). The external Ringer's solution contained 125 mM NaCl, 2.5 mM KCl, 2 mM CaCl_2_, 1 mM MgCl_2_, 26 mM NaHCO_3_, 1.25 mM NaH_2_PO_4_, 10 mM D-glucose, and 0.1 mM picrotoxin (to inhibit GABAergic synapses) and was bubbled with 95% O_2_ and 5% CO_2_ at room temperature (24°C). To evoke parallel fiber-evoked excitatory postsynaptic currents (PF-EPSCs), a stimulating electrode was placed in the molecular layer (stimulus intensity, 0 to 200 µA, 10 µs duration), and selective stimulation of parallel fibers (PFs) was confirmed by the paired-pulse facilitation (PPF) of PF-EPSC amplitudes with a 50 ms interstimulus interval. The current traces were filtered at 1 kHz and digitized at 4 kHz.

## Results

CAPS2 exon 3-skipped homozygote mice (CAPS2^Δex3/Δex3^) were born and exhibited no obvious difference in life expectancy compared to control mice [22]. However, the body weight of CAPS2^Δex3/Δex3^ male mice was lower than that of their wild-type littermates (34.21±0.97 g in wild types *vs*. 28.12±0.4 g in CAPS2^Δex3/Δex3^ mice at 4 months old, mean ± s.e.m., *P*<0.01, by Student's *t*-test).

### CAPS2-dex3 protein fails to accumulate in parallel fibers, axons of cerebellar granule cells, in the molecular layer of the cerebellar cortex

**Figure 1:**
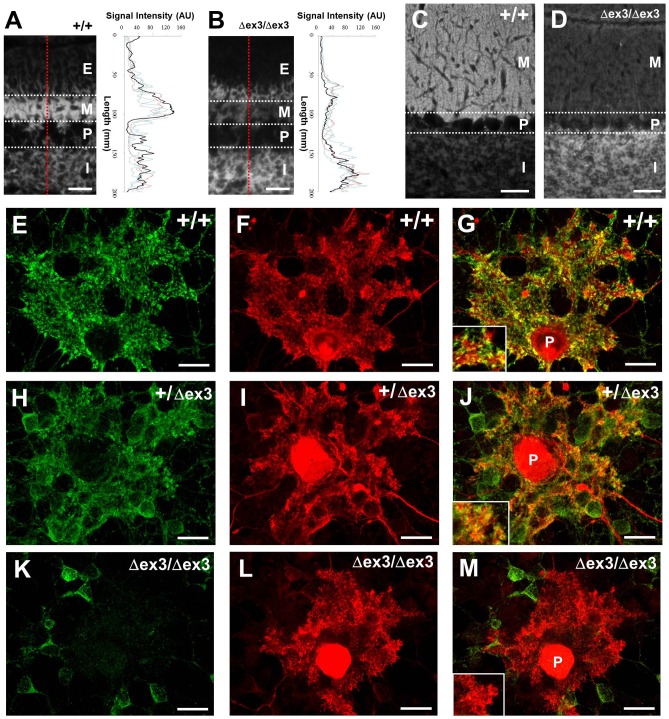
Distribution of CAPS2 protein in the CAPS2^Δex3/Δex3^ mouse cerebellum and cerebellar culture. (A, B) Sagittal sections of P7 wild-type (A) and CAPS2^Δex3/Δex3^ (B) cerebellum were immunolabeled with anti-CAPS2 antibody. Immunosignal intensity of red dotted lines is shown by black lines in the graphs. Signal intensity from the other three images is shown in colored lines. Scale bars, 30 µm. (C, D) Sagittal sections of P21 wild-type (C) and CAPS2^Δex3/Δex3^ (D) cerebellum were immunolabeled with an anti-CAPS2 antibody. E, external granular layer; M, molecular layer; P, Purkinje cell layer; I, internal granular layer. Scale bars, 20 µm. (E–M) Subcellular localization of CAPS2 (green; E, H, K) and calbindin (red; F, I, L) in wild-type (E–G), CAPS2^+/Δex3^ (H–J) and CAPS2^Δex3/Δex3^ (K–M) cerebellar primary cultures at 14 days *in vitro* (DIV). A merged image is shown in (G, J, M). Insets: higher magnification. P, Purkinje cell. Scale bars, 20 µm.

At first, we examined the subcellular localization of CAPS2-deletion of exon3 (dex3) expressed in CAPS2^Δex3/Δex3^ mice. Development of the cerebellar cortex includes dynamic changes in the laminar architecture. The external granular layer (EGL), the site at which robust proliferation of granule cell precursors occurs, thickens progressively until a peak is reached at approximately the first postnatal week; the EGL then gradually thins as the proliferation of these precursors decline, disappearing by the weaning stage, which occurs around the third postnatal week. In the P7 cerebellum, wild-type CAPS2 protein was mostly localized in the granule cell axons (parallel fibers) extending into the molecular layer (ML) (Fig. 1A), whereas dex3 protein was not localized in the axons in the ML and instead accumulated densely in the cell soma in the internal granule cell layer (IGL) and sparsely in the EGL (Fig. 1B). This symmetric localization pattern was more drastic in the P21 cerebellum: wild-type CAPS2 protein was localized in the ML, axon of cerebellar granule cells (Fig. 1C), whereas dex3 protein was not in the ML but accumulated in the IGL, soma and/or dendrites of cerebellar granule cells (Fig. 1D).

We further analyzed the subcellular localization of CAPS2-dex3 protein using cerebellar primary cultures at 21 days *in vitro* (DIV) (Fig. 1E–M). In wild-type cultures, punctate immunoreactivity for CAPS2 was largely concentrated near Purkinje cells innervated by many granule cell axons, demonstrating the presynaptic localization of CAPS2 in parallel fibers (PFs) (Fig. 1E–G). In contrast, in CAPS2^Δex3/Δex3^ cultures, CAPS2-immunoreactive signal was hardly detected around calbindin-immunopositive dendrites of Purkinje cells and predominated in soma of granule cells instead (Fig. 1K–M). In heterozygote CAPS2^+/Δex3^ cultures, CAPS2-immunoreactive signal was localized in granule cell soma as well as around the dendrites of Purkinje cells (Fig. 1H–J).

Taken together, the results obtained using an animal model showed that the exon 3-skipped alternative splicing affects the subcellular localization of CAPS2 protein in the axonal compartment.

### CAPS2-dex3 affects axonal localization and release of BDNF and NT-3 in cerebellar granule cells

**Figure 2:**
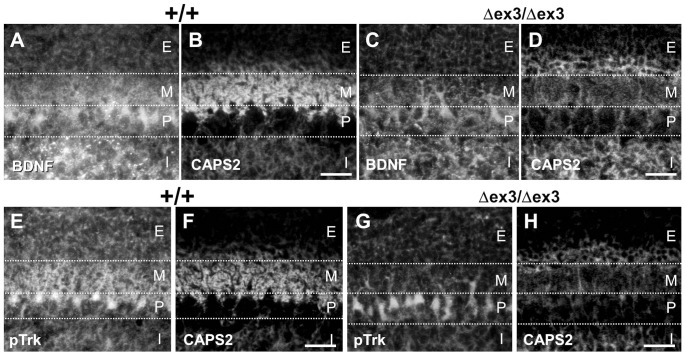
Decreased immunoreactivity of BDNF and phosphorylated Trk (Tyr490) in the molecular layer of CAPS2^Δex3/Δex3^ cerebellum. (A–D) Sagittal sections of P7 wild-type (A, B) and CAPS2^Δex3/Δex3^ (C, D) cerebellum were immunolabeled with an anti-BDNF (A, C) and an anti-CAPS2 (B, D) antibody. (E–H) Sagittal sections of P7 wild-type (E, F) and CAPS2^Δex3/Δex3^ (G, H) cerebellum were immunolabeled with an anti-pTrk (E, G) and an anti-CAPS2 (F, H) antibody. E, external granular layer; M, molecular layer; P, Purkinje cell layer; I, internal granular layer. Scale bars, 40 µm.

CAPS2 is found associated with secretory vesicles containing BDNF and NT-3, and it promotes release of these two neurotrophins from cerebellar granule cells [9]. TrkB, the BDNF receptor, is expressed in postsynaptic Purkinje cells as well as in presynaptic granule cells. BDNF is released from the PF terminals of granule cells, which innervate the distal dendrites of Purkinje cells, and is thought to function in autocrine and anterograde signaling [27], [28]. BDNF is also known to be released from climbing fibers that innervate the proximal dendrites of Purkinje cells, and the trafficking of BDNF along climbing fibers is dependent on another family member, CAPS1 [29]. We examined the subcellular localization of endogenous BDNF in CAPS2^Δex3/Δex3^ mice. In the P7 wild-type cerebellum, BDNF immunoreactivity was localized in the ML, IGL, and the soma and distal and proximal dendrites of Purkinje cells (Fig. 2A). In contrast, in the CAPS2^Δex3/Δex3^ cerebellum, BDNF immunoreactivity was detected in the proximal dendrites of Purkinje cells, but seemed to be reduced in the ML (PF terminals) (Fig. 2C).

Secreted neurotrophins activate their Trk receptors on target cells, leading to the autophosphorylation of Trk receptors. To localize Trk receptors activated by neurotrophins released from granule cells, we next immunostained the cerebellar cortex of P7 wild-type and CAPS2^Δex3/Δex3^ mice with an anti-phosphorylated Trk (pTrk) antibody (Fig. 2E and G). In the wild-type, intense pTrk immunoreactivity was observed in the ML and the Purkinje cell layer (Fig. 2E). The immunoreactivity in the wild-type ML was observed around both distal and proximal dendrites of Purkinje cells (reflecting Trk activation by anterograde signaling), although a part of this labeling was probably derived from the parallel fiber terminals (reflecting Trk activation by autocrine signaling). In comparison, in CAPS2^Δex3/Δex3^ mice, pTrk immunoreactivity was decreased in the ML, but detectable at a similar level in the soma, compared with wild-type (Fig. 2G). These results suggest that, in the cerebellar cortex of P7 CAPS2^Δex3/Δex3^ mice, there is a reduction in autocrine and paracrine activation of Trk by BDNF released from parallel fibers. It is probable that Trk activation in the Purkinje cell soma was dependent on BDNF released from climbing fiber terminals.

**Figure 3:**
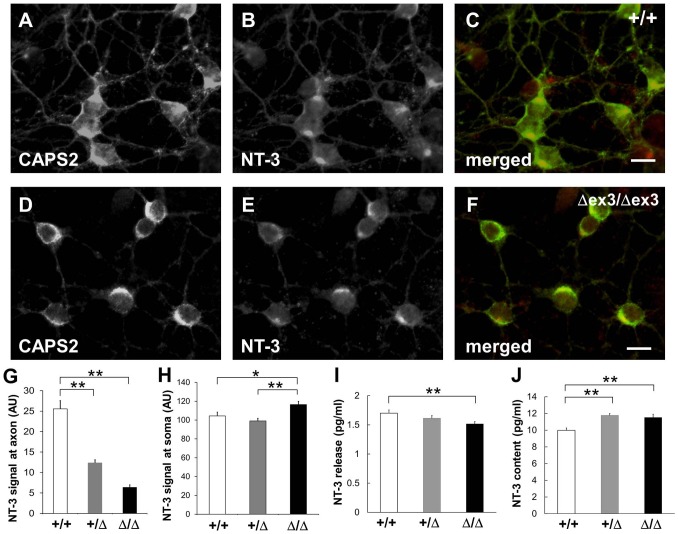
Decreased trafficking and release of NT-3 in the CAPS2^Δex3/Δex3^ primary cerebellar culture. (A–F) Subcellular localization of CAPS2 (A, D) and NT-3 (B, E) in wild-type (A–C) and CAPS2^Δex3/Δex3^ (D–F) cerebellar primary cultures at 7 DIV. A merged image of CAPS2 (*green*) and NT-3 (*red*) is shown in (C, F). Scale bars, 10 µm. (G) Immunolabeling intensities of NT-3 along the axons of wild-type (white bar, *n* = 27), CAPS2^+/Δex3^ (gray bar, *n* = 23) and CAPS2^Δex3/Δex3^ (black bar, *n* = 21) cells are shown. Axons were identified by tau immunostaining and by their characteristic thin and long processes. Signal intensities were quantified with NIH ImageJ per unit length (arbitrary unit). (H) Immunolabeling intensities of NT-3 on the somata of wild-type (white bar, *n* = 23), CAPS2^+/Δex3^ (gray bar, *n* = 23) and CAPS2^Δex3/Δex3^ (black bar, *n* = 22) cells are shown. Signal intensities were quantified per unit area (arbitrary unit). *P<0.05; **P<0.01, by Student's *t*-test. (I) NT-3 release activity in the wild-type (white bar, *n* = 16), CAPS2^+/Δex3^ (gray bar, *n* = 21) and CAPS2^Δex3/Δex3^ (black bar, *n* = 27) cerebellar cultures was evaluated by measuring the levels of NT-3 spontaneously secreted into the culture medium by primary dissociation cultures at 7 DIV with an enzyme immunoassay. *P*<0.05, one-factor ANOVA. **P<0.01, by post-hoc *t*-test. (J) The amount of NT-3 in the cell lysates of wild-type (white bar, *n* = 7), CAPS2^+/Δex3^ (gray bar, *n* = 13) and CAPS2^Δex3/Δex3^ (black bar, *n* = 7) cultures was evaluated as indicated in (I). *P*<0.01, one-factor ANOVA. **P<0.01, by post-hoc *t*-test. The error bars indicate the s.e.m.

We next examined subcellular localization of NT-3 and quantified amounts of released NT-3 and cellular NT-3 in primary cultured cerebellar granule cells at 7DIV. In wild-type cerebellar sections at P7, the CAPS2 protein produced in granule cells is localized in both the internal granule layer and the molecular layer, in which soma and axons of granule cells, respectively, are localized (Fig. 1A). In contrast, at P21, the protein is almost exclusively localized in the molecular layer, in which the axon terminals of granule cells innervate the dendrites of Purkinje cells (Fig. 1C). Similarly, in wild-type cerebellar cultures at 7 DIV, CAPS2 immunoreactivity was localized not only in axons, but in soma of granule cells as well (Fig. 3A). NT-3 immunoreactivities were also localized in neurite meshwork as well as soma throughout the wild-type cell cultures (Fig. 3A–C). On the other hand, such meshwork-like neurite immunoreactivities for CAPS2 and NT-3 were decreased in CAPS2^Δex3/Δex3^ cell cultures (Fig. 3D–F) compared with wild-type cultures. There was a significant difference in NT-3 immunolabeling intensity along the axon between wild-type and dex3 neurons (Fig. 3G). In addition, NT-3 immunolabeling intensity was increased in the somata of dex3 neurons compared with wild-type neurons (Fig. 3H).

We next analyzed the amounts of NT-3 released into cerebellar culture media. Endogenous NT-3, activity-dependently released into the media, was barely detectable in cultures by enzyme-linked immunoabsorbent assay [9]. We compared the total amount of NT-3 released into the culture media over a period of 7 days, during which a fraction of the NT-3 is likely released by spontaneous activity, between wild-type and mutant mouse cultures. As shown in Fig. 3I, the amount of NT-3 in the media was significantly reduced in CAPS2^Δex3/Δex3^ cultures compared with wild-type cultures. On the contrary, the amount of NT-3 in the cell lysate was increased in CAPS2^+/Δex3^ and CAPS2^Δex3/Δex3^ cultures as compared with wild-type cultures (Fig. 3J). Thus, it might be assumed that release of NT-3 was decreased and intracellular accumulation of NT-3 was increased instead in CAPS2^Δex3/Δex3^.

**Figure 4:**
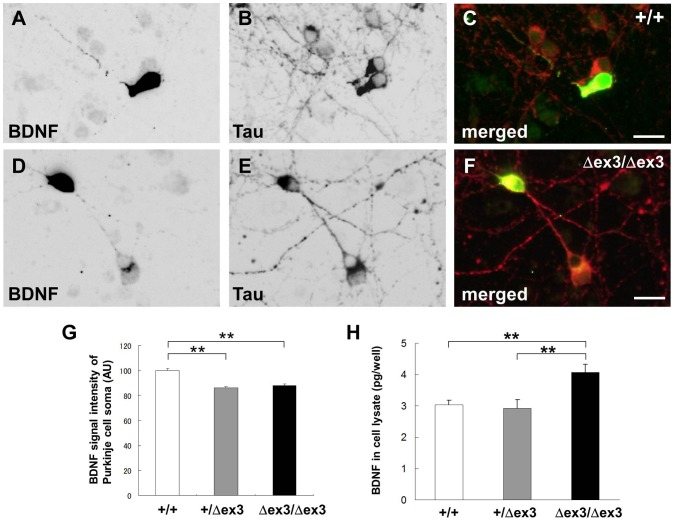
Aberrant distribution of BDNF and decreased BDNF immunoreactivity in the cerebellum. (A–F) Subcellular localization of exogenously expressed C-terminal HA-tagged BDNF (A, D) and tau protein (B, E) in wild-type (A–C) and CAPS2^Δex3/Δex3^ (D–F) cerebellar primary cultures immunostained for HA and tau at 8 DIV. A merged image of HA (*green*) and tau (*red*) is shown in (C, F). Scale bars, 20 µm. (G) The intensity of BDNF immunoreactivity in the Purkinje cells of wild-type (white bar, *n* = 226), CAPS2^+/Δex3^ (gray bar, *n* = 215) and CAPS2^Δex3/Δex3^ (black bar, *n* = 215) cultures was quantified using confocal microscopy. *P*<0.01, one-factor ANOVA. **P<0.01, by post-hoc *t*-test. (H) The amount of BDNF in the cell lysates of wild-type (white bar, *n* = 6), CAPS2^+/Δex3^ (gray bar, *n* = 8) and CAPS2^Δex3/Δex3^ (black bar, *n* = 6) cultures was evaluated at 7 DIV with an enzyme immunoassay. *P*<0.01, one-factor ANOVA. **P<0.01, by post-hoc *t*-test. The error bars indicate the s.e.m.

Next, we tested subcellular localization of HA-tagged BDNF (BDNF-HA) that was exogenously overexpressed in cerebellar granule cell cultures. HA immunoreactivity was localized to axons with tau immunoreactivity in wild-type granule cells (Fig. 4A–C) but was yet very little in axons of CAPS2^Δex3/Δex3^ granule cells (Fig. 4D–F). The amount of BDNF released into the culture media was hardly detected, even if BDNF was exogenously transfected as previously described [9], [24]. Therefore, to compare BDNF release levels between wild-type and CAPS2^Δex3/Δex3^ cultures, we evaluated immuno-intensity of endogenous BDNF incorporated into target Purkinje cell bodies after released from granule cell axons using confocal microscopy (Fig. 4G). The result showed that internalization of BDNF into the Purkinje cell soma was significantly reduced in CAPS2^Δex3/Δex3^ granule cells compared with wild-type cells. On the other hand, amount of cellular BDNF contents was higher in CAPS2^Δex3/Δex3^ cell lysates than wild-type cell lysates (Fig. 4H). Together, these data suggest that BDNF is accumulated in the cell soma and its release might be affected, when expression of exon 3-skipped CAPS2 is increased.

### Developmental deficits in the postnatal cerebellum of CAPS2^Δex3/Δex3^ mice

**Figure 5:**
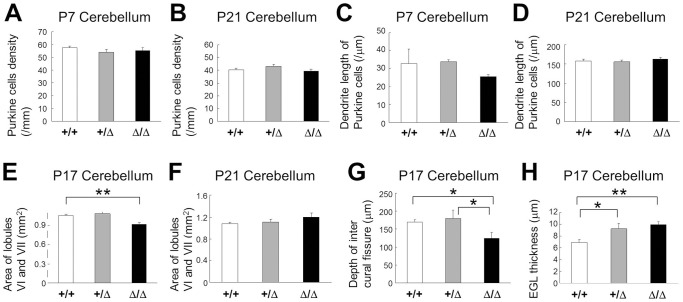
Abnormal immunohistochemistry in the CAPS2^Δex3/Δex3^ cerebellum. (A) The cell densities of calbindin-positive Purkinje cells for wild-type (white, *n* = 8), CAPS2^+/Δex3^ (gray, *n* = 9), and CAPS2^Δex3/Δex3^ (black, *n* = 10) mice at P7. (B) The cell densities of calbindin-positive Purkinje cells for wild-type (white, *n* = 8), CAPS2^+/Δex3^ (gray, *n* = 10), and CAPS2^Δex3/Δex3^ (black, *n* = 10) mice at P21. (C) The dendrite length of calbindin-positive Purkinje cells for wild-type (white, *n* = 24), CAPS2^+/Δex3^ (gray, *n* = 30), and CAPS2^Δex3/Δex3^ (black, *n* = 30) mice at P7. (D) The dendrite length of calbindin-positive Purkinje cells for wild-type (white, *n* = 9), CAPS2^+/Δex3^ (gray, *n* = 10), and CAPS2^Δex3/Δex3^ (black, *n* = 9) mice at P21. (E) The area of lobules VI and VII of wild-type (white, *n* = 10), CAPS2^+/Δex3^ (gray, *n* = 8), and CAPS2^Δex3/Δex3^ (black, *n* = 12) mice at P17. *P*<0.01, one-factor ANOVA. (F) The area of lobules VI and VII of wild-type (white, *n* = 8), CAPS2^+/Δex3^ (gray, *n* = 8), and CAPS2^Δex3/Δex3^ (black, *n* = 8) mice at P21. (G) The depth of intercrural fissure between lobules VI and VII of wild-type (white, *n* = 10), CAPS2^+/Δex3^ (gray, *n* = 6), and CAPS2^Δex3/Δex3^ (black, *n* = 9) mice at P17. *P*<0.05, one-factor ANOVA. (H) The thickness of the EGL of wild-type (white, *n* = 12), CAPS2^+/Δex3^ (gray, *n* = 7), and CAPS2^Δex3/Δex3^ (black, *n* = 15) mice at P17. *P*<0.05, one-factor ANOVA. The error bars indicate the s.e.m. *P<0.05; **P<0.01, by post-hoc *t*-test.

We analyzed morphological development of CAPS2^Δex3/Δex3^ cerebellum. In terms of Purkinje cells, there was no significant difference in the cell density among three genotypes wild-type, CAPS2^+/Δex3^, and CAPS2^Δex3/Δex3^ at P7 (Fig. 5A) and P21 (Fig. 5B). Moreover, there was no difference of Purkinje cell dendrite length among three genotypes at P7 and P21 (Fig. 5C and D). In terms of cerebellar lobulation, the area of vermian lobules VI-VII was decreased in CAPS2^Δex3/Δex3^ cerebellum at P17 (Fig. 5E) but not P21 (Fig. 5F) as compared with wild-type. The depth of the intercrural fissure which divides the lobule VI and VII was decreased in CAPS2^Δex3/Δex3^ cerebellum as compared with wild-type and CAPS2^+/Δex3^ (Fig. 5G). In terms of the EGL in which granule cell precursors robustly proliferate by the second postnatal period, CAPS2^+/Δex3^ and CAPS2^Δex3/Δex3^ cerebella had thicker EGL than wild-type even at P17 (Fig. 5H). But, the EGL was no longer detectable in the CAPS2^Δex3/Δex3^ cerebellum by P28 (data not shown). Taken together, these data suggest that postnatal CAPS2^Δex3/Δex3^ cerebellum appears to have a developmental delay similarly to CAPS2 knockout (KO) cerebellum [24].

**Figure 6:**
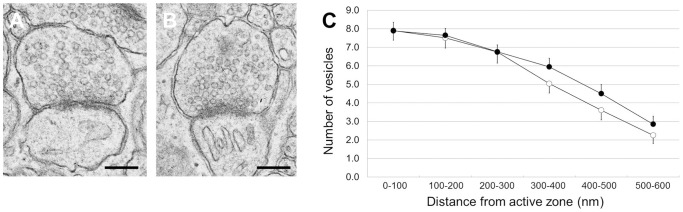
Distribution of vesicles within PF terminals. (A, B) Electron micrographs of a PF-PC synapse in the P21 wild-type cerebellum (A) and another in the CAPS2^Δex3/Δex3^ cerebellum (B). Scale bars, 400 nm. (C) Quantification of vesicle distribution on the electron micrographs. The distance between vesicles and the active zone is indicated on the x-axis; numbers of vesicles of wild-type (open circles; *n* = 51) and CAPS2^Δex3/Δex3^ (closed circles; *n* = 54) are represented on the y-axis. The error bars indicate the s.e.m.

Next, we analyzed the fine structure of PF-Purkinje cell (PC) synapses at P21. In the wild-type cerebellum, synaptic vesicles within the PF terminals connecting Purkinje cell spines were concentrated near the active zones and were fully distributed over the presynaptic boutons of PFs (Fig. 6A). Overall synaptic cytoarchitecture regarding clear synaptic vesicles, active zone and postsynaptic density appeared to be similar between wild-type (Fig. 6A) and CAPS2^Δex3/Δex3^ (Fig. 6B). Quantification of presynaptic vesicle distribution from the active zone showed that number of synaptic vesicle 300–600 nm far from the active zone tended to be larger in CAPS2^Δex3/Δex3^ than wild-type (Fig. 6C), although there was no statistical difference between these two genotypes.

### Impairment of short-term plasticity at CAPS2^Δex3/Δex3^ PF-PC synapses

**Figure 7:**
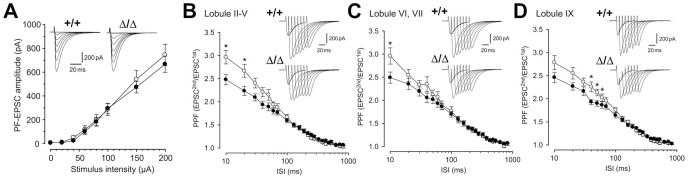
Impairment of short-term synaptic plasticity at PF-PC synapses in the CAPS2^Δex3/Δex3^ mouse cerebellum. (A) Plots showing the relationship between the PF-EPSC amplitude and stimulus intensity applied to PFs in CAPS2^Δex3/Δex3^ mice (closed circles; *n* = 12) and wild-type littermates (open circles; *n* = 12), at P25–35. Insets show representative EPSC traces evoked by PF stimuli of different intensities. (B–D) The mean paired-pulse ratios recorded from each lobule of P19–20 cerebellar slices were plotted against various interstimulus intervals. Recordings from lobules II–V (wild-type, *n* = 14; CAPS2^Δex3/Δex3^, *n* = 14), VI–VII (wild-type, *n* = 12; CAPS2^Δex3/Δex3^, *n* = 16), and IX (wild-type, *n* = 13; CAPS2^Δex3/Δex3^, *n* = 13) are shown in (B), (C), and (D), respectively. Insets show representative traces obtained from CAPS2^Δex3/Δex3^ mice and wild-type littermates. V_h_ = −80 mV. The error bars indicate the s.e.m. *P<0.05, by the Mann–Whitney *U* test.

We investigated the synaptic function of PF-PC synapses at P25–35. Little difference was found to exist between wild-type and CAPS2^Δex3/Δex3^ mice in terms of the peak amplitudes of PF-evoked excitatory postsynaptic currents (PF-EPSCs) (Fig. 7A), thus indicating that the basic synaptic transmission function in CAPS2^Δex3/Δex3^ mice was unimpaired.

Paired-pulse facilitation (PPF) of the PF-EPSC (defined as the ratio of the amplitude of the second EPSC to that of the first EPSC) is thought to reflect changes in presynaptic function and is considered to be a kind of short-term synaptic plasticity [30]. We performed PPF analysis at PF-PC synapses in the anterior lobe (lobules II–V), central lobe (lobules VI–VII), and posterior lobe (lobule IX) at P25–35 (Fig. 7B). The results revealed that, for brief interstimulus intervals (<100 ms), the degree of PPF at PF-PC synapses was markedly lower in CAPS2^Δex3/Δex3^ mice than in wild-type mice. Impairment of this presynaptic function was observed in all three lobes examined. However, synaptic plasticity at PF-PC synapses, such as a long-term depression (LTD), a molecular basis of the motor learning and memory [31], was normally observed in CAPS2^Δex3/Δex3^ mice as well as wild-type littermates (data not shown). The results suggest that expression of dex3 disturbs the presynaptic properties of PF-PC synapses.

## Discussion

In this report, we focused on the cellular and physiological phenotype in the cerebellum of a mouse line CAPS2^Δex3/Δex3^ expressing dex3, the same as a rare alternatively-spliced variant of human CAPS2 that was identified in some individuals with autism [14]. We verified a deficit in axonal localization of dex3 protein in cerebellar granule cells in *in vitro* cultures as well as *in vivo*. Thus, we assume that CAPS2^Δex3/Δex3^ mice have disturbance in CAPS2-mediated promotion of the secretory vesicle secretion pathway: probably decreased axonal promotion but normal somato-dendritic promotion. Interestingly, CAPS2^Δex3/Δex3^ mice also displayed a decrease in axonal localization of BDNF and NT-3. Release of BDNF and NT-3 from cerebellar granule cells was decreased in CAPS2^Δex3/Δex3^ mice. In connection with the indispensable role of these two neurotrophins in cerebellar postnatal development, CAPS2^Δex3/Δex3^ mice showed several developmental deficits including decreased area of vermian lobules VI and VII, shallow intercrural fissures, delayed proliferation of granule cell precursors in the EGL, and delayed migration of post-mitotic granule cells. At PF-PC synapses, paired-pulse facilitation was also impaired in CAPS2^Δex3/Δex3^ mice. CAPS2 is thought to promote BDNF and NT-3 release [9], [24]. Our results suggest subcellular locality in CAPS2-mediated enhancement of BDNF and NT-3 release is associated with the proper development and function of the cerebellum.

### Specific lobule hypoplasia and neurotrophin

We showed that the area of lobules VI-VII and the depth of intercrural fissure which divide lobule VI and VII were decreased in the CAPS2^Δex3/Δex3^ cerebellum. BDNF and NT-3 KO mice also show a lobulation deficit between lobules VI and VII [27], [32]. Katoh-Semba and her colleagues showed a higher concentration of NT-3 in posterior lobules than in anterior lobules, and that depletion of NT-3 from the mouse brain causes an increase in granule cell apoptosis within lobules VI-VII [25], suggesting that the requirement of neurotrophins for granule cell survival seems to be different between cerebellar lobules. In this regard, it is noteworthy that there are localized high BDNF protein levels in lobules VI-VII and localized high NT-3 mRNA levels in lobules VI-VII during development [24]. Taken together, these results suggest that lobules VI-VII express more BDNF and NT-3 than do the other cerebellar compartments during development; thus, these lobules are more susceptible to the effects of depletion of these neurotrophins.

### PF-PC synapse function of CAPS2^Δex3/Δex3^ cerebellum

We found the degree of PPF at PF-PC synapses was lower in CAPS2^Δex3/Δex3^ mice, which seemed to be consistent in understanding a role of CAPS2 in PFs, because similar results were obtained in CAPS2 KO mice (Sadakata et al., 2007a). In addition, a concomitant decrease in PPF during enhancement of evoked EPSCs is generally accepted to be a strong indication for presynaptic modifications of synaptic transmission. BDNF KO mice is known to show impaired PPF at PF-PC synapses together with increased vesicle number per synapse [33]. In this regard, it is interesting the number of synaptic vesicle far from the active zone tended to be larger in CAPS2^Δex3/Δex3^ PF terminal. It is probable that decrease PPF of CAPS2^Δex3/Δex3^ mice depends on the change of synaptic vesicle distribution by decreased BDNF release.

### The possible effect of CAPS2-dex3 copy number

The amount of BDNF released into the culture media was barely detectable, even when BDNF was exogenously transfected. Therefore, to compare BDNF release levels between wild-type and CAPS2^Δex3/Δex3^ cultures, we evaluated the immunolabeling intensities of endogenous BDNF incorporated into target Purkinje cell bodies using confocal microscopy. In Fig. 4G, CAPS2^Δex3/Δex3^ cultures showed a decrease in endogenous BDNF uptake into Purkinje neurons. Correspondingly, as shown in Fig. 4H, there was an increase in BDNF levels in their lysates, where most of the BDNF was likely derived from granule cells. In comparison, CAPS2^+/Δex3^ cultures showed a decrease in endogenous BDNF uptake into Purkinje neurons, while there was no difference in BDNF levels in their lysates. With the available data, it is difficult to make definitive statements on BDNF dynamics in the different cultures, because one must consider multiple factors; i.e., BDNF produced and transported in granule cells, that released from granule cells, and that internalized by endocytosis into Purkinje cells via binding to TrkB receptors. In addition, a change in BDNF-TrkB signaling may affect the normal development and function of Purkinje cells and granule cells, even in CAPS2^+/Δex3^ animals. It is possible that there is a decrease in BDNF receptors in CAPS2^+/Δex3^ Purkinje cells compared with wild-type Purkinje cells. The effect of CAPS2-dex3 copy number on BDNF production, trafficking, release and endocytosis remain to be elucidated in future studies.

### CAPS2 gene disruption and autistic phenotypes

In this report, we focused on the cerebellar phenotypes of CAPS2^Δex3/Δex3^ mice and found that CAPS2^Δex3/Δex3^ mice show the cerebellar phenotypes described above. Some of these phenotypes are similar with those observed in autistic patients. Abnormal reduction in lobules VI and VII of the cerebellar vermis is a characteristic morphological deficit reported in the brains of autistic patients [34], and hypoplasia of these lobules was also suggested to relate the abnormal exploratory behavior of autism [35]. It is of interest that decreased PPF was also shown in a mouse model of Rett syndrome [36], which is considered to be an autistic spectrum disorder. Taken together, our findings are suggestive of an association of the increased exon 3 splicing of CAPS2 in the cerebellar deficits of autistic patients.
